# Biological activities and volatile constituents of *Daucus muricatus* L. from Algeria

**DOI:** 10.1186/1752-153X-6-48

**Published:** 2012-05-30

**Authors:** Amel Bendiabdellah, Mohammed El Amine Dib, Nassim Djabou, Hocine Allali, Boufeldja Tabti, Alain Muselli, Jean Costa

**Affiliations:** 1 Laboratoire des Substances Naturelles et Bioactives, Université de Tlemcen, BP 119, 13000, Tlemcen, Algérie; 2 UMR CNRS 6134, Laboratoire Chimie des Produits Naturels, Campus Grimaldi, Université de Corse, BP 52, 20250, Corte, France

**Keywords:** *Daucus muricatus*. L, Essential oils, HS-SPME, GC/MS, Antimicrobial and antioxidant activities

## Abstract

**Background:**

In order to find new bioactive natural products, the antimicrobial and antioxidant activities of essential oil components extracted from the separated organs of the Algerian medicinal and aromatic plant *Daucus muricatus* L. were studied.

**Results:**

The chemical composition of essential oils obtained by hydrodistillation (HD) was investigated using Gas Chromatography–Retention Indices (GC-RI) and GC–Mass Spectrometry (GC-MS). Two types of essential oils were produced by *D. muricatus*: (i) The oil from roots is mainly composed by nonterpenic oxygenated compounds (59.8 g/100 g), and (ii) the aerial part oils (i.e., the leaves, stems, flowers, and umbels) was mainly composed by terpenic hydrocarbon compounds (62.3–72.2 g/100 g). The chemical composition of the volatile fraction isolated from different organs of *Daucus muricatus* were studied by HS–SPME/GC–RI and GC–MS after optimization of Solid Phase MicroExtraction parameters. For all organs studied, the main volatiles emitted by the plant were hydrocarbon compounds (60.7–82.2 g/100 g). Only quantitative differences between the volatiles of the separated organs studied were observed. In addition, the activity of the oil of *D. muricatus* against eight bacterial strains and one yeast was investigated. The oil from roots revealed active against *S. aureus*, while the essential oil obtained from the aerial parts was active against the yeast *C. albicans*.

**Conclusions:**

*Daucus muricatus* essential oil seems be a promising source of natural products with potential antimicrobial activity.

## Background

*Daucus* is a genus belonging to the Apiaceae family and consists of about 600 species that are widely distributed around the world. In Algeria, the *Daucus* genus is represented by more than 27 species living in dry and uncultivated areas [1], and they are mostly found from Tlemcen to Mascara [1,2]. The most prevalent of the species is *Daucus carota* L. (carrot) reported with eight subspecies throughout Algeria [1]. *Daucus muricatus* L., synonym of *Artedia muricata* L., *Caucalis muricata* L., and *Platyspermum muricatum* Hoffm., is widely distributed in Algeria, Spain, Portugal, Corsica, Sardinia, Sicily, Italy, the Aegean Islands, and Turkey [2]. *Daucus muricatus* is an annual plant 30–50 cm high, dark green, bristling at the base, with a stem thickened at the nodes and branches spreading erect. The leaves are soft and lanceolate in their periphery in segments cut into narrow strips with white flowers. The umbels opposite the leaves at the end are contracted, the fruit are large, elliptical and compressed, armed with spines expanded and confluent at the base [1,2]. Several investigations deal with the chemical composition of essential oils of the *Daucus* species [3-27]. While no study has investigated *D. muricatus* essential oils, most of them have reported the chemical composition of essential oils from *D. carota* and its subspecies [3,4,6-16,20,22,23,25-27]. However, only three studies have reported the chemical composition of essential oils from *Daucus* species from Algeria. The first reported the chemical composition of the essential oil of *D. reboudii* Coss. [17], and the other two reported the chemical composition of the oil from *D. crinitus* Desf. [18,19]. Previous reports showed that the chemical composition of *Daucus* species was more dominated by monoterpene hydrocarbon compounds such α-pinene and sabinene [3,4,10,14,15], and occasionally by phenylpropanoids compounds such as apiol, myristicin, and isochavicol [3,14-16]. Several studies recently investigated the biological activity of *Daucus* essential oils [6,10,12,19,20]. However, there remain many species and subspecies of *Daucus* that have not yet been examined.

As part of our ongoing chemical investigation of the essential oils from the Algerian *Daucus* genus [18] and our search for active natural products to fight nosocomial infections, we investigated for the first time the chemical composition and biological activities of *Daucus* muricatus L. through the study of: (i) the volatile components of *D. muricatus* roots, leaves, stems, flowers, and umbels extracted by hydrodistillation and by solid phase microextraction (SPME) using gas chromatography (GC) and gas chromatography–mass spectrometry (GC–MS), (ii) the antibacterial activity of *D. muricatus* essential oil against nine species of microorganisms involved in nosocomial infections using paper disc diffusion and agar dilution methods.

## Results and discussion

### Essential oil chemical composition

GC–RI and GC–MS analysis of *D. muricatus* essential oils obtained from the roots, stems, leaves, flowers, and umbels that accounted for 92.8, 94.7, 94.5, 95.4, and 95.7 g/100 g of the oils, respectively and allowed the identification of 99 compounds. Their retention indices, yields, and relative concentrations expressed in g/100 g of essential oil are shown in Table 1. Among these, 39 monoterpenes, 32 sesquiterpenes, 22 nonterpenic compounds, three diterpenes, two phenylpropanoids, and one C_13_-isopropenoid were identified. All components were identified by comparison of their EI–MS and GC-retention indices with those of our laboratory-produced “Arômes” library, with the exception of nine components that were identified by comparison with spectral data and retention indices from the literature (see Table 1). Two types of essential oils were produced by *D. muricatus*. The root oils are mainly composed by oxygenated compounds (59.8 g/100 g), and the aerial part oils (i.e., the leaves, stems, flowers, and umbels) were dominated by the occurrence of hydrocarbon compounds (62.3–72.2 g/100 g). The main components of root oil were nonterpenic aliphatic compounds that accounted for 56.7 g/100 g, such as eicosane (18.6 g/100 g), undecan-2-one (10.2 g/100 g), and tridecanol (6.4 g/100 g). Conversely, the main components of aerial organs of D. *muricatus* were monoterpene hydrocarbons (52.0–58.5 g/100 g). For all organs studied, limonene (21.9–24.0 g/100 g) and α-pinene (9.9–21.8 g/100 g) were the main components. Their relative abundances were followed by that of sabinene (4.7–8.1 g/100 g) in stems, leaves and flowers, and trans-sabinyl acetate in umbels (12.1 g/100 g). On moving from the bottom to the top of the plant, we noted that the relative concentrations of nonterpenic compounds decreased as follows: 56.7 g/100 g in the roots, 12.5 g/100 g in the stems, 7.7 g/100 g in the leaves, 4.0 g/100 g in the flowers, and then 3.6 g/100 g in the umbels.

**Table 1:** **Composition of the essential oils of*D. muricatus*(roots, leaves, stems, flowers and umbels)**

						**Separated organs**^**e**^					
**N°**	**Components**^**a**^	**lRIa**^**b**^	**RIa**^**c**^	**RIp**^**d**^	**Aerial parts**	**Stems**	**Leaves**	**Flowers**	**Umbels**	**Roots**	**Identification**^**f**^
1	Nonane	900	898	899	1.0	0.5	1.3	1.1	0.3	-	RI, MS
2	α-Thujene	932	923	1011	0.6	tr	0.2	0.7	0.1	-	RI, MS
3	α- Pinene	936	931	1016	16.7	9.9	18.9	14.3	21.8	0.5	RI, MS
4	Camphene	950	944	1056	0.3	0.2	0.2	0.2	0.8	tr	RI, MS
5	Thuja-2,4 (10) diene	946	945	1085	tr	0.2	0.1	tr	-	-	RI, MS
6	Sabinene	973	967	1111	18.9	5.1	4.7	8.1	4.6	0.2	RI, MS
7	β-pinene	978	970	1102	2.5	2.1	1.8	2.8	1.4	0.1	RI, MS
8	Myrcene	987	980	1152	1.8	2.1	2.3	1.6	2.6	-	RI, MS
9	α-Phellandrene	1002	997	1140	0.6	0.5	0.4	0.6	0.2	-	RI, MS
10	α-Terpinene	1013	1008	1158	1.1	1.1	1.1	1.5	0.8	0.1	RI, MS
11	p-Cymene	1015	1012	1147	1.3	4.4	1.4	0.9	1.4	0.1	RI, MS
12	Limonene	1025	1022	1195	14.2	22.6	21.3	24.0	21.9	0.3	RI, MS
13	(Z)-β-Ocimene	1029	1025	1215	0.1	0.1	0.1	tr	0.2	tr	RI, MS
14	γ-Terpinene	1051	1049	1239	2.6	2.4	1.6	3.1	1.1	tr	RI, MS
15	trans-Sabinene hydrate	1053	1052	1438	0.2	0.1	0.1	0.3	tr	-	RI, MS
16	Nonan-2-one	1070	1073	1392	0.1	0.6	0.1	tr	0.1	0.3	RI, MS
17	p-Cymenene	1075	1077	1420	0.6	0.7	0.1	tr	tr	0.1	RI, MS
18	Terpinolene	1082	1079	1292	0.3	0.6	0.3	0.7	0.2	0.1	RI, MS
19	Linalool	1083	1085	1392	0.1	tr	0.4	0.3	0.2	-	RI, MS
20	Undecane	1100	1100	1098	0.6	2.1	1.9	0.4	0.9	0.1	RI, MS
21	Limonene-1,2-epoxide	1117	1119	1446	0.2	0.2	0.2	0.3	0.4	0.9	RI, MS
22	trans-Pinocarveol	1126	1120	1632	0.3	0.7	0.1	0.2	0.2	0.2	RI, MS
23	trans-2-Nonenal	1135	1133	1525	0.2	1.2	0.4	0.1	0.2	1.1	RI, MS
24	Pinocarvone	1137	1135	1520	0.1	0.4	0.1	0.1	tr	0.1	RI, MS
25	Borneol	1150	1149	1670	0.1	0.2	0.1	tr	0.1	tr	RI, MS
26	Cryptone	1160	1159	1642	0.2	0.5	0.3	0.1	0.3	0.2	RI, MS
27	Terpinen-4-ol	1164	1160	1563	2.7	1.1	0.8	3.1	0.8	1.7	RI, MS
28	Decan-2-one	1176	1170	1503	0.1	0.8	0.3	0.2	0.1	0.2	RI, MS
29	α-Terpineol	1176	1177	1685	0.2	0.3	0.1	0.1	tr	tr	RI, MS
30	Myrtenol	1178	1182	1763	tr	0.5	0.1	0.1	0.2	0.7	RI, MS
31	Decanal	1188	1185	1481	tr	0.8	0.1	0.1	tr	0.2	RI, MS
32	Dodecane	1200	1199	1201	tr	0.2	0.1	tr	0.1	0.3	RI, MS
33	Citronellol	1213	1211	1724	0.1	0.1	0.1	tr	-	tr	RI, MS
34	Carvone	1214	1215	1749	tr	0.2	0.1	tr	-	tr	RI, MS
35	Pulegone	1215	1216	1602	tr	0.1	tr	tr	-	-	RI, MS
36	p-Anisaldehyde	1218	1219	2049	tr	0.6	tr	tr	0.3	-	RI, MS
37	Geraniol	1235	1235	1799	0.1	0.1	0.1	0.2	-	tr	RI, MS
38	trans-Myrtanol	1240	1236	1858	tr	0.1	0.2	0.2	0.5	-	RI, MS
39	*cis*-Chrysanthenyl acetate	1253	1242	1548	0.3	2.2	0.5	tr	0.1	0.2	RI, MS
40	α-Terpinen-7-al	1257	1256	1763	0.1	0.2	0.2	0.4	0.3	tr	RI, MS
41	Thymol	1267	1264	2149	tr	0.1	0.1	0.1	-	0.6	RI, MS
42	Bornyl acetate	1270	1266	1536	0.2	0.6	0.2	0.1	0.4	0.9	RI, MS
43	Undecan-2-one	1273	1270	1579	0.3	3.9	0.5	0.3	0.8	10.2	RI, MS
44	*trans*-Sabinyl acetate	1278	1271	1650	2.6	1.5	0.7	0.2	12.1	3.1	RI, MS, Ref1
45	Carvacrol	1278	1278	2224	tr	0.2	0.4	tr	0.1	0.5	RI, MS
46	Undecan-2-ol	1287	1285	1723	0.1	0.3	0.1	tr	-	1.2	RI, MS
47	Myrtenyl acetate	1332	1320	1701	0.1	0.1	0.2	0.1	tr	0.3	RI, MS
48	δ-Elemene	1340	1337	1535	0.2	0.5	1.7	0.1	0.2	-	RI, MS
49	Geranyl acetate	1362	1360	1715	tr	0.2	tr	2.3	-	0.2	RI, MS
50	Undecanol	1363	1365	1820	0.2	0.6	1.1	1.2	0.1	0.3	RI, MS
51	α-Copaene	1379	1377	1488	0.2	0.6	0.5	0.2	0.5	-	RI, MS
52	*β*-Bourbonene	1379	1383	1496	tr	0.4	0.3	0.2	0.1	-	RI, MS
53	*β*-Elemene	1389	1390	1570	0.3	0.3	0.1	0.2	0.5	-	RI, MS
54	Dodecanal	1389	1395	1673	tr	0.1	0.1	0.2	tr	1.9	RI, MS
55	Aristolene	1418	1420	1553	0.1	tr	0.1	0.3	0.1		RI, MS
56	trans-Caryophyllene	1424	1422	1586	1.8	0.6	3.8	2.4	2.1	0.3	RI, MS
57	Geranyl acetone	1429	1426	1842	0.1	0.4	0.2	0.3	0.4	0.1	RI, MS
58	*β*-Copaene	1430	1430	1579	tr	0.1	0.1	tr	0.4	-	RI, MS
59	α-Humulene	1455	1450	1655	0.4	0.4	0.5	0.3	0.4	0.5	RI, MS
60	*β*-Ionone	1468	1460	1902	0.5	0.2	0.1	0.8	0.4	-	RI, MS
61	Dodecanol	1472	1468	1754	0.2	0.2	0.2	tr	0.1	3.7	RI, MS
62	γ-Muurolene	1473	1471	1667	0.1	0.9	0.3	0.1	-	-	RI, MS
63	Germacrene D	1479	1476	1665	1.5	0.2	1.6	1.4	2.9	1.5	RI, MS
64	*trans*-β-Bergamotene	1480	1475	1598	tr	tr	tr	0.1	tr	0.8	RI, MS
65	6-epi-Shyobunone	1481	1480	1855	0.1	0.1	0.1	0.2	0.3	0.3	RI, MS
66	γ-Humulene	1483	1487	1682	0.2	0.4	0.2	0.4	0.1	-	RI, MS
67	Bicyclogermacrene	1494	1490	1706	0.4	0.4	0.3	0.2	0.2	-	RI, MS
68	α-Muurolene	1496	1498	1710	0.4	0.2	0.3	0.4	0.2	-	RI, MS
69	Shyobunone	1500	1501	1897	0.1	0.5	0.5	0.4	0.2	0.1	RI, MS
70	δ-Cadinene	1520	1515	1715	0.8	0.2	0.6	0.6	1.7	0.5	RI, MS
71	E-α-Bisabolene	1531	1526	1733	0.6	0.8	1.6	0.4	1.2	0.2	RI, MS
72	Isochavicol isobutyrate	1541	1538	2136	5.3	1.2	2.2	6.7	1.6	2.3	RI, MS
73	Germacrene B	1552	1555	1794	0.2	0.6	0.2	0.2	0.8	tr	RI, MS, Ref1
74	1,5-Epoxy-salvial-4(14)-ene	1561	1561	1903	0.1	0.7	0.4	0.5	0.4	3.6	RI, MS
75	Spathulenol	1572	1564	2091	0.5	1.2	1.4	0.3	0.6	-	RI, MS
76	Caryophyllene oxide	1578	1582	1943	tr	0.1	0.3	0.1	tr	0.5	RI, MS
77	Tridecanol	1580	1586	2034	-	tr	tr	tr	tr	6.4	RI, MS, Ref2
78	Viridiflorol	1590	1586	2071	0.1	0.9	0.3	0.5	0.2		RI, MS
79	Copaborneol	1592	1595	2142	0.5	0.8	0.3	1.5	0.1	1.4	RI, MS
80	Guaia-6,10(14)-diene-4β-ol	1610	1609	2119	1.1	2.5	2.1	6.6	0.5	0.9	RI, MS, Ref1
81	epi-Cubenol	1621	1623	2046	0.2	0.3	0.2	0.4	0.3	0.9	RI, MS
82	Cubenol	1630	1631	2001	0.5	0.2	0.2	0.3	tr	tr	RI, MS
83	τ-Muurolol	1633	1635	2156	0.5	0.7	0.3	0.6	0.7	1.4	RI, MS
84	α-Cadinol	1643	1644	2212	0.5	0.7	0.5	0.6	0.3	0.9	RI, MS
85	Isochavicol 2-methyl butyrate	1651	1654	2256	0.1	0.3	0.2	0.2	-	0.4	RI, MS
86	(Z)-α-Santalol	1669	1665	2306	0.1	0.4	0.1	0.1	0.1	-	RI, MS
87	Eudesma-4(15),7-dien-1β-ol	1671	1672	2346	0.1	0.3	0.2	0.1	0.5	1.3	RI, MS
88	(E,Z)-Farnesol	1685	1680	2313	tr	0.2	0.1	0.1	0.1	3.1	RI, MS
89	Heptadecane	1700	1699	1698	tr	0.3	0.3	0.1	0.1	0.5	RI, MS
90	Tetradecanoic acid	1761	1756	2651	tr	0.1	0.2	0.2	-	1.7	RI, MS, Ref2
91	Neophytadiene	1807	1806	1920	0.1	0.2	1.7	0.1	0.1	1.3	RI, MS, Ref1
92	Diisobutyl ester	1826	1826	2525	0.2	0.1	0.4	tr	tr	0.9	RI, MS, Ref2
93	6,10,14-Trimethylpentadecanone	1845	1842	2125	0.2	0.1	0.4	tr	0.1	1.8	RI, MS, Ref2
94	Hexadecanoic acid	1951	1956	2821	0.1	0.2	tr	tr	0.2	3.1	RI, MS, Ref1
95	Eicosane	2000	2000	1998	tr	0.1	tr	tr	0.2	18.6	RI, MS
96	(Z)-Phytol	2080	2085	2611	0.2	0.2	0.8	0.1	0.3	-	RI, MS
97	(E)-Phytol	2114	2119	2568	0.3	0.2	1.9	tr	0.1	-	RI, MS
98	Tricosane	2300	2302	2299	0.1	0.2	tr	tr	0.1	1.5	RI, MS
99	Pentacosane	2500	2498	2501	0.1	0.1	0.2	0.1	0,2	2.7	RI, MS
	Total identification g/100g				90.9	93.7	93.6	98.7	95.3	90.1	
	Essential oil yield% (w/w)				0.2	0.04	0.03	0.09	0.12	0.02	
	Monoterpene hydrocarbons				61.6	52,0	54.5	58.5	57.1	1.5	
	Oxygenated monoterpenes				8.2	10.2	5.3	9.2	16.5	9.1	
	Sesquiterpene hydrocarbons				7.2	6.6	12.2	7.5	11.4	3.8	
	Oxygenated sesquiterpenes				4.4	9.6	7,0	12.3	4.3	14.4	
	Phenylpropanoids				5.4	2.2	2.5	7,0	1.9	3.3	
	Oxygenated diterpenes				0.5	0.4	2.7	0.1	0.4	-	
	Diterpenes hydrocarbons				0.1	0.2	1.7	0.1	0.1	1.3	
	Non-terpenic compounds				3.5	12.5	7.7	4,0	3.6	56.7	

a Order of elution is given on apolar column (Rtx-1), b Retention indices of literature on the apolar column (lRIa) reported from König et al., 2001.,c Retention indices on the apolar Rtx-1 column (RIa), d Retention indices on the polar Rtx-Wax column (RIp), e Quantification was carried out using RFs relative to tridecane as internal standard, g/100 g: concentration expressed in g/100 g of essential oil are given on the apolar column except for components with identical RIa (concentrations are given on the polar column), tr = trace (<0,05 g/100 g), f RI: Retention Indices; MS: Mass Spectrometry in electronic impact mode; Ref1,: compounds identified from literature data König et al., 2001, Ref2,: compounds identified from literature data NIST Chemistry WebBook.

### HS-SPME analysis

The volatiles emitted from the *D. muricatus* roots, leaves, stems, umbels, and flowers were investigated using HS–SPME under optimized parameters. The optimization of the HS–SPME sampling parameters was conducted using fresh plant material based on the sum of the total peak areas obtained using GC–FID. The maximum sum of the total peak area was acquired for an equilibrium and extraction temperature of 70°C, an equilibrium time of 60 min, and an extraction time of 30 min. The GC–RI and GC–MS analysis identified 78 components: 42 monoterpenes, 18 nonterpenic compounds, 16 sesquiterpenes, and two phenylpropanoids (Table 2). Identification of 74 components was conducted by comparing their EI–MS and retention indices with those in our laboratory-produced “Arômes” library, and four components were identified by comparing their EI–MS data and their apolar retention indices with those reported in the literature and in commercial libraries. Regarding the organ contribution to the aromatic plant fingerprint, it should be noted that the volatile constituents were more abundant in the leaves than in the other parts of the plant. Our analysis showed that the chemical composition of the HS fractions obtained from different organs was qualitatively similar but differed by the relative amounts of the main components. Relative to *D. muricatus* oil, the main volatiles emitted by plant were hydrocarbon compounds (60.7–82.2%) for all organs studied. More precisely, the sum of hydrocarbon monoterpenes (33.6%–64.1%) and hydrocarbon nonterpenic compounds (6.4%–25.5%) was higher than that of oxygenated compounds, which never accounted for more than 23.9 g/100 g. The main volatile components of roots were terpinolene (10.2%), bornyl acetate (9.7%), p-cymene (9.1%), α-pinene (8.7%), and undecane (7.2%). The relative amounts of terpinolene (0.4%–1.3%) and p-cymene (3.1%–3.4%) were lower in the aerial organs, and the main volatile emitted by leaves, flowers and umbels was limonene (30.6%, 19.1%, and 28.1%, respectively). In the stems, limonene (13.3%) was present in lower amounts than undecane (16.9%), which was identified as a major component. In addition, undecane was produced in appreciable amounts in leaves, flowers, and umbels (6.1%, 3.6%, and 7.4%, respectively). They were accompanied by α-pinene, which accounted for 8.1% in stems and always more than 13.1% in leaves, flowers and umbels. With these hydrocarbon compounds, we noted the occurrence of trans-sabinyl acetate, which had a relatively higher concentration in umbels (9.6%) than in the other aerial organs (2.6%–3.8%). The chemical differences observed between the essential oils and the volatile fractions extracted using HD and SPME, respectively, can be explained by the fact that the first technique is based on the liquid quasi-total extraction of plant volatiles, and the latter technique is controlled by a solid/gas equilibrium step. During hydrodistillation, the most volatile and water soluble compounds are lost in the gaseous phase and in the hydrolate, respectively, whereas, with HS extraction, it is the fiber affinity of each compound that monitors the sampling of the volatiles. As a consequence, it should be noted that 23 compounds (1a–1e, 5a, 8a, 9a, 13a, 14a, 19a, 20a, 21a, 22a, 22b, 27a, 27b, 29a, 38a, 47a, 51a, 54a, and 65a) were only identified in the volatile fractions extracted using HS–SPME.

**Table 2:** **Chemical composition of ***D. muricatus* volatile fractions extracted by HS-SPME

					**Separated organs**^**e**^				
**N°**	**Components**^**a**^	**lRIa**^**b**^	**RIa**^**c**^	**Aerial parts**	**Stems**	**Leaves**	**Flowers**	**Umbels**	**Roots**
1a	Heptane	700	700	2.6 ± 0.07	7.9 ± 0.28	1.3 ± 0.11	1.1 ± 0.15	0.3 ± 0.04	0.2 ± 0.01
1b	3-Methyl butanol	709	705	0.3 ± 0.01	0.7 ± 0.01	0.2 ± 0.01	0.4 ± 0.01	0.2 ± 0.01	0.1 ± 0.01
1c	3-Methyl-pentan-2-ol	754	760	0.4 ± 0.14	0.4 ± 0.01	0.2 ± 0.01	0.5 ± 0.09	0.4 ± 0.01	0.7 ± 0.01
1d	Hexanal	780	771	0.2 ± 0.01	0.4 ± 0.01	0.1 ± 0.01	0.1 ± 0.01	0.3 ± 0.06	1.4 ± 0.07
1	Nonane	900	898	0.5 ± 0.07	0.5 ± 0.02	1.1 ± 0.22	0.4 ± 0.01	tr	0.1 ± 0.01
1e	Artemisiatriene	923	921	0.2 ± 0.01	-	-	0.3 ± 0.06	0.1 ± 0.01	-
2	α-Thujene	932	923	0.1 ± 0.01	-	0.1 ± 0.01	0.5 ± 0.04	0.1 ± 0.01	-
3	α-Pinene	936	931	13.2 ± 0.74	8.1 ± 0.36	16.1 ± 0.89	13.1 ± 0.97	15.5 ± 1.1	8.7 ± 0.14
4	Camphene	950	944	0.2 ± 0.01	tr	0.1 ± 0.01	0.3 ± 0.01	0.3 ± 0.08	0.4 ± 0.01
5a	Butyl butyrate	970	966	0.2 ± 0.01	-	0.2 ± 0.01	0.2 ± 0.01	0.2 ± 0.01	0.1 ± 0.01
6	Sabinene	973	967	2.9 ± 0.21	1.4 ± 0.14	2.3 ± 0.45	6.5 ± 0.33	1.5 ± 0.46	0.3 ± 0.01
7	β-Pinene	978	970	0.9 ± 0.06	0.7 ± 0.02	1.2 ± 0.56	1.1 ± 0.51	1.1 ± 0.13	1.6 ± 0.21
8	Myrcene	987	980	2.9 ± 0.13	1.5 ± 0.14	3.2 ± 0.21	2.9 ± 0.12	3.9 ± 0.66	0.1 ± 0.01
8a	Yomogi alcohol	991	981	0.4 ± 0.07	0.7 ± 0.01	0.4 ± 0.01	0.4 ± 0.01	0.4 ± 0.05	-
9	α-Phellandrene	1002	997	1.5 ± 0.13	-	3.1 ± 0.16	2 ± 0.29	1.1 ± 0.29	-
9a	3-Carene	1010	1005	0.1 ± 0.01	-	0.1 ± 0.01	0.1 ± 0.01	0.1 ± 0.01	0.4 ± 0.01
10	α-Terpinene	1013	1008	0.8 ± 0.06	-	0.3 ± 0.01	2.8 ± 0.22	0.2 ± 0.01	0.1 ± 0.01
11	p-Cymene	1015	1012	3.2 ± 0.32	3.4 ± 0.12	3.1 ± 0.51	3.4 ± 0.51	3.2 ± 0.11	9.1 ± 0.76
12	Limonene	1025	1022	22.4 ± 1.28	13.3 ± 0.56	30.6 ± 0.99	19.1 ± 0.77	28.1 ± 0.82	6.9 ± 0.35
13	(Z)-β-Ocimene	1029	1025	0.2 ± 0.01	0.4 ± 0.01	0.1 ± 0.01	0.2 ± 0.02	0.1 ± 0.01	0.3 ± 0.01
13a	(E)-β-Ocimene	1041	1031	0.1 ± 0.01	0.1 ± 0.01	0.2 ± 0.02	0.2 ± 0.01	0.1 ± 0.01	0.3 ± 0.01
14	γ-Terpinene	1051	1049	3.2 ± 0.09	4.1 ± 0.54	2.5 ± 0.22	5.1 ± 0.55	1.3 ± 0.35	1.2 ± 0.01
14a	Octanol	1063	1052	0.3 ± 0.01	tr	0.6 ± 0.01	0.4 ± 0.08	0.3 ± 0.05	-
16	Nonan-2-one	1070	1073	0.6 ± 0.07	0.7 ± 0.02	0.5 ± 0.01	0.6 ± 0.08	0.6 ± 0.09	0.2 ± 0.01
17	p-Cymenene	1075	1077	0.3 ± 0.06	-	0.2 ± 0.01	0.4 ± 0.06	0.7 ± 0.03	3.1 ± 0.13
18	Terpinolene	1082	1079	0.7 ± 0.06	0.6 ± 0.01	0.9 ± 0.07	1.3 ± 0.23	0.4 ± 0.01	10.2 ± 0.86
19	Linalool	1083	1085	1.4 ± 0.08	3.2 ± 0.28	0.5 ± 0.01	1.1 ± 0.29	0.9 ± 0.1	0.2 ± 0.01
19a	α-Thujone	1089	1086	0.5 ± 0.04	1.8 ± 0.09	tr	0.5 ± 0.02	0.4 ± 0.02	0.1 ± 0.01
20	Undecane	1100	1100	8.9 ± 0.76	16.9 ± 0.89	6.1 ± 0.38	3.6 ± 0.14	7.4 ± 0.69	7.2 ± 0.26
20a	3-Octyl acetate	1113	1103	1.8 ± 0.12	0.7 ± 0.06	0.2 ± 0.01	0.3 ± 0.01	0.2 ± 0.01	0.2 ± 0.01
21a	Camphor	1123	1119	1.1 ± 0.07	2.9 ± 0.16	0.7 ± 0.01	0.6 ± 0.06	0.2 ± 0.01	0.5 ± 0.01
22	trans-Pinocarveol	1126	1120	0.4 ± 0.01	0.7 ± 0.01	0.3 ± 0.02	0.4 ± 0.01	0.6 ± 0.02	0.1 ± 0.01
22a	Citronellal	1129	1130	0.1 ± 0.01	tr	0.1 ± 0.01	0.1 ± 0.01	0.4 ± 0.05	-
22b	cis-Verbenol	1132	1132	0.1 ± 0.01	-	0.1 ± 0.01	0.1 ± 0.01	0.1 ± 0.01	-
24	Pinocarvone	1137	1135	0.1 ± 0.01	-	0.1 ± 0.01	0.1 ± 0.01	0.2 ± 0.02	0.7 ± 0.01
25	Borneol	1150	1149	0.5 ± 0.08	0.9 ± 0.01	0.4 ± 0.01	0.3 ± 0.04	0.4 ± 0.01	-
26	Cryptone	1160	1159	0.5 ± 0.06	0.5 ± 0.01	0.5 ± 0.08	0.4 ± 0.01	0.7 ± 0.09	3.8 ± 0.11
27	Terpinene-4-ol	1164	1160	1.4 ± 0.18	2.1 ± 0.43	0.9 ± 0.06	1.8 ± 0.36	1.1 ± 0.15	0.1 ± 0.01
27a	Myrtenal	1172	1163	0.1 ± 0.01	tr	0.1 ± 0.01	0.1 ± 0.01	0.1 ± 0.01	0.3 ± 0.02
27b	Estragole	1175	1169	1.1 ± 0.09	1.1 ± 0.58	1.3 ± 0.29	1.7 ± 0.12	0.6 ± 0.08	-
29	α-Terpineol	1176	1177	0.4 ± 0.05	0.5 ± 0.04	0.4 ± 0.08	0.4 ± 0.03	0.5 ± 0.01	0.2 ± 0.01
29a	Verbenone	1184	1178	0.3 ± 0.01	0.7 ± 0.01	0.1 ± 0.01	0.2 ± 0.01	0.1 ± 0.01	-
31	Decanal	1188	1185	0.1 ± 0.01	-	0.1 ± 0.01	0.1 ± 0.01	0.2 ± 0.01	-
33	Citronellol	1213	1211	1.1 ± 0.14	1.1 ± 0.22	1.1 ± 0.33	0.9 ± 0.06	1.5 ± 0.23	0.1 ± 0.01
34	Carvone	1214	1215	0.7 ± 0.07	1.1 ± 0.09	0.5 ± 0.01	0.6 ± 0.04	0.6 ± 0.02	0.1 ± 0.01
35	Pulegone	1215	1216	0.3 ± 0.01	0.8 ± 0.02	0.2 ± 0.01	0.2 ± 0.01	0.1 ± 0.01	-
37	Geraniol	1235	1235	0.2 ± 0.01	tr	0.1 ± 0.01	0.4 ± 0.02	0.1 ± 0.01	0.8 ± 0.05
38	trans-Myrtanol	1240	1236	0.1 ± 0.01	0.1 ± 0.01	tr	0.1 ± 0.01	0.3 ± 0.01	-
38a	Geranial	1244	1239	0.1 ± 0.01	tr	0.1 ± 0.01	0.1 ± 0.01	tr	-
39	cis-Chrysanthenyl acetate	1253	1242	0.1 ± 0.01	tr	0.1 ± 0.01	tr	0.1 ± 0.01	-
42	Bornyl acetate	1270	1266	0.6 ± 0.05	1.2 ± 0.11	0.4 ± 0.05	0.5 ± 0.06	0.6 ± 0.04	9.7 ± 0.55
43	Undecan-2-one	1273	1270	0.6 ± 0.08	0.8 ± 0.04	0.6 ± 0.08	0.4 ± 0.03	0.5 ± 0.01	0.3 ± 0.01
44	trans-Sabinyl acetate	1278	1271	5.1 ± 0.47	3.8 ± 0.26	2.6 ± 0.12	3.5 ± 0.38	9.6 ± 0.53	
47a	Neryl acetate	1342	1335	2.0 ± 0.31	2.4 ± 0.36	3.4 ± 0.65	1.3 ± 0.35	0.9 ± 0.1	0.3 ± 0.01
48	δ-Elemene	1340	1337	0.3 ± 0.02	0.7 ± 0.06	0.3 ± 0.05	0.1 ± 0.01	tr	0.1 ± 0.01
49	Geranyl acetate	1362	1360	0.1 ± 0.01	tr	tr	0.2 ± 0.01	tr	0.2 ± 0.01
50	Undecanol	1363	1365	0.1 ± 0.01	tr	tr	tr	tr	1.9 ± 0.01
51	α-Copaene	1379	1377	0.7 ± 0.01	1.1 ± 0.12	0.4 ± 0.02	0.6 ± 0.03	0.9 ± 0.08	0.4 ± 0.01
51a	Daucene	1380	1379	0.5 ± 0.02	0.9 ± 0.08	0.1 ± 0.01	0.2 ± 0.01	0.7 ± 0.01	0.7 ± 0.06
54	Dodecanal	1389	1395	0.2 ± 0.01	0.3 ± 0.01	0.1 ± 0.01	0.1 ± 0.01	0.1 ± 0.01	1.3 ± 0.01
54a	Tetradecane	1400	1399	0.1 ± 0.01	0.2 ± 0.02	0.1 ± 0.01	0.1 ± 0.01	tr	-
56	*trans*-Caryophyllene	1424	1422	2.3 ± 0.23	1.4 ± 0.13	3.1 ± 0.25	3.8 ± 0.26	1.2 ± 0.15	1.9 ± 0.18
58	*β*-Copaene	1430	1430	0.3 ± 0.02	0.4 ± 0.01	0.6 ± 0.05	0.3 ± 0.01	0.3 ± 0.01	0.4 ± 0.01
59	α-Humulene	1455	1450	0.4 ± 0.05	-	0.1 ± 0.01	0.9 ± 0.08	0.1 ± 0.01	0.1 ± 0.01
61	Dodecanol	1472	1468	tr	tr	tr	tr	tr	3.4 ± 0.28
62	γ-Muurolene	1473	1471	1.3 ± 0.13	0.5 ± 0.01	0.6 ± 0.01	1.5 ± 0.42	1.9 ± 0.21	4.3 ± 0.16
63	Germacrene D	1479	1476	0.4 ± 0.04	0.5 ± 0.06	0.2 ± 0.01	0.4 ± 0.02	0.3 ± 0.01	0.2 ± 0.01
64	*trans*-β-Bergamotene	1480	1475	tr	tr	tr	tr	tr	0.5 ± 0.03
65	6-epi-Shyobunone	1481	1480	0.1 ± 0.01	tr	0.1 ± 0.01	0.2 ± 0.01	0.2 ± 0.01	-
65a	β-Selinene	1486	1481	0.2 ± 0.01	0.2 ± 0.01	0.2 ± 0.02	0.1 ± 0.01	0.2 ± 0.03	0.5 ± 0.02
69	Shyobunone	1500	1501	0.1 ± 0.01	tr	0.1 ± 0.01	0.1 ± 0.01	0.1 ± 0.01	0.7 ± 0.01
70	δ-Cadinene	1520	1515	0.6 ± 0.04	0.5 ± 0.01	0.3 ± 0.04	0.1 ± 0.01	1.3 ± 0.16	0.9 ± 0.07
71	(*E*)-α-Bisabolene	1531	1526	1.1 ± 0.31	0.5 ± 0.02	1.4 ± 0.12	0.9 ± 0.1	1 ± 0.45	0.4 ± 0.01
72	Isochavicol isobutyrate	1541	1538	0.3 ± 0.05	tr	0.3 ± 0.01	0.9 ± 0.06	0.1 ± 0.01	0.1 ± 0.1
76	Caryophyllene oxide	1578	1582	0.2 ± 0.01	-	0.1 ± 0.01	0.4 ± 0.01	0.1 ± 0.01	0.7 ± 0.01
77	Tridecanol	1580	1586	tr	tr	tr	tr	tr	1.1 ± 0.07
87	Eudesma-4(15),7-dien-1β-ol	1671	1672	tr	-	tr	tr	tr	0.6 ± 0.01
89	Heptadecane	1700	1699	0.4 ± 0.13	-	1.3 ± 0.65	0.2 ± 0.01	0.2 ± 0.01	-
	Total identification (%)			97.8	95.4	99.1	94.3	97.6	90.6
	GC-FID Total area 10^5^			103	22	154	108	107	68
	Monoterpene hydrocarbons			52.9	33.6	64.1	59.3	57.8	42.8
	Oxygenated monoterpenes			20.6	25.6	14.4	16	20.5	17.6
	Sesquiterpene hydrocarbons			8.1	5.8	7.2	8.7	7.2	10.4
	Oxygenated sesquiterpenes			0.4	-	0.3	0.7	0.4	2.0
	Phenylpropanoids			0.3	-	0.3	0.9	0.1	0.1
	Non-terpenic compounds			15.5	30.4	12.8	8.7	11.6	17.7

^a^ Order of elution is given on apolar column (Rtx-1). Numbers correspond to those in Table 1. The volatile components identified exclusively from the HS-fractions were affected by a letter, b Retention indices of literature on the apolar column (lRIa) reported from König et al., 2001., c Retention indices on the apolar Rtx-1 column (RIa), d Percentages (means of three analyses) obtained by GC-FID (on RTX-1: apolar column) under optimized HS-SPME parameters: temperature: 70°C, equilibrium time: 120 min; extraction time: 30 min.

### Antimicrobial activity (assay disk)

Preliminary screening of the antimicrobial activity in vitro of the essential oils from *D. muricatus* species against nine pathogenic microorganisms were studied using the filter paper disc agar-diffusion technique. The results showed variation in the antimicrobial properties of the plant essential oil (Table 3). The essential oil showed strong activity (inhibition zone >20 mm), moderate activity (inhibition zone <20–12 mm), and no inhibition (zone <12 mm). The highest activity (diameter of inhibition zone 22 mm) was demonstrated against *S. aureus* by the essential oil of the root, while the lowest (diameter of inhibition zone 6 mm) was demonstrated against E. coli by oil from the aerial parts. Other hand, *C. albicans, B. cereus* and *L. monocytogenes* were also prone to growth inhibition with diameter zones of inhibition ranging from 12 to 16 mm. Rest of the bacterial strains (*B. subtilis*, *E. faecalis*, *P. aeruginosa*, *K. pneumonia* and *E. coli*) showed no inhibition, with diameter of zones of inhibition ranging from 8 to 10 mm (Table 3).

**Table 3:** **Antimicrobial activity of ***D. muricatus* essential oil

**Microorganisms**	**Disc diffusion assay (mm)**		**MIC (μg/ml)**	
	**Roots**	**Aerial parts**	**Roots**	**Aerial parts**
**Gram-positive bacterium**				
*B. subtilis*	9	8	> 6000	> 6000
*L. monocytogenes*	14	13	65	250
*B. cereus*	15	12	65	250
*S. aureus*	22	10	8	> 6000
*P. aeruginosa*	9	10	> 6000	> 6000
*E. faecalis*	10	8	> 6000	> 6000
**Gram-negative bacterium**				
*K. pneumoniae*	9	8	> 6000	> 6000
*E. coli*	8	6	> 6000	> 6000
**Yeast**				
*C. albicans*	12	16	95	45

### Minimum inhibitory concentrations (MIC)

The in-vitro antibacterial activities of essential oil from the roots and aerial parts of *D. muricatus* against the employed bacteria were assessed qualitatively and quantitatively by the presence or absence of inhibition zones. The noted antibacterial and antifungal effects of the two are presented in Table 3. In general, the roots oil showed higher activity against bacteria than oil of aerial parts. The most prominent inhibitory action of roots oil was observed against *S. aureus* with a MIC of 0.8 μg/ml. However, *B. cereus* and *L. monocytogenes* showed moderate activity with MIC values of 65 μg/ml. As for the antifungal effect, the aerial parts oil was found to be effective against the pathogenic yeast *C. albicans* (MIC = 45 μg/ml) and an average activity antimicrobial on the *B. cereus* and *L. monocytogenes* with a MIC of 250 μg/ml. It should be noted that the highest tested concentration (6000 μg/ml) of had no effect on other growth of microorganisms. Various chemical compounds isolated by hydrodistillation of oils from *D. muricatus* have direct activity against many species of bacteria, such as terpenes and a variety of aliphatic hydrocarbons (alcohols, aldehydes and ketones). The lipophilic character of their hydrocarbon skeleton and the hydrophilic character of their functional groups are of main importance in the antimicrobial action of essential oils components. Therefore, a rank of activity has been proposed as follows: phenols > aldehydes > ketones > alcohols > esters > hydrocarbons [28]. The activity of the roots oil could be explained at least partially by its content of undecan-2-one (10.2%). This ketone was previously proved to have antimicrobial and nematicidal activity [29,30]. The higher activity of the roots oil compared to the aerial parts oil could be attributed to this fact. Another major class of this oil, aliphatic alcohols was, likewise, previously reported as an antimicrobial compound and was reported to possess strong to moderate activities against several bacteria [31].

## Conclusion

Volatiles isolated from separated organs of *D. muricatus* by HS–SPME and hydrodistillation were investigated using GC–RI and GC–MS. Concerning the essential oils, oil from *D. muricatus* roots was mainly composed of oxygenated compounds, while oil from aerial parts (i.e., the leaves, stems, flowers, and umbels) was dominated by hydrocarbon compounds. Moreover, the study of the volatiles sampled by HS–SPME showed that the chemical composition of the HS fractions obtained from different organs was qualitatively similar but differed by the relative concentrations of the main components. It is interesting to note that the sample preparation method impacted quantitatively on the GC profile of *D. muricatus* volatiles. The antimicrobial properties of *D. muricatus* essential oils tested on nine microorganisms species showed that oil from roots was active against *S. aureus*, while essential oil obtained from aerial parts was active against the yeast *C. albicans*.

## Experimental

### Plant material and oil isolation

Separated organs (stems, leaves, flowers, umbels and roots) from *D. muricatus* were collected in Bensekrane forest area (North West of Tlemcen, Algeria) [287 m, 35 °07′N 1 °22′O] on September 2009. Voucher specimens were deposited in the herbarium of the Tlemcen University Botanical Laboratory (Voucher number: UBL 128.09). A portion of each organ was stored at 4°C for eventual further studies. The oils were isolated by hydrodistillation (400–450 g of plant per sample) for 6 h using a Clevenger-type apparatus [32] according to the European Pharmacopoeia and yielded 0.02% for roots and 0.03-0.12% for aerial parts w/w of oil.

### HS-SPME conditions

The single organs of *D. muricatus* (stems, leaves, flowers, umbels and roots separately) were cut roughly with scissors (1–2 cm long) before subjection to HS-SPME. The SPME device (Supelco) coated with divinylbenzene/carboxen/polydimethylsiloxane (DVB/CAR/PDMS, 30 μm) was used for extraction of the plant volatiles. Optimization of conditions was carried out using fresh organs of the plant (1 g in a 20 mL vial) and based on the number and the sum of total peak areas measured on GC-FID. Temperature, equilibration time and extraction time were selected after nine experiments combining four temperatures (30, 50, 70 and 90°C), four equilibration times (20, 40, 60 and 80 min) and three extraction times (15, 30 and 45 min). After sampling, SPME fibre was inserted into the GC and GC-MS injection ports for desorption of volatile components (5 min), both using the splitless injection mode. Before sampling, each fibre was reconditioned for 5 min in the GC injection port at 260°C. HS-SPME and subsequent analyses were performed in triplicate. The coefficient of variation (1.6% < CV < 17.8%) calculated on the basis of total area obtained from the FID-signal for the samples indicated that the HS-SPME method produced reliable results.

### Gas chromatography

GC analyses were carried out using a Perkin–Elmer (Waltham, MA, USA) Autosystem XL GC apparatus equipped with a dual flame ionization detection system and a fused-silica capillary columns (60 m x 0.22 mm I.D., film thickness 0.25 μm), Rtx-1 (polydimethylsiloxane). The oven temperature was programmed from 60°C to 230°C at 2°C/min and then held isothermally at 230°C for 35 min. Injector and detector temperatures were maintained at 280°C. Samples were injected in the split mode (1/50), using helium as the carrier gas (1 mL/min); the injection volume was 0.2 μL. Retention indices (RI) of the compounds were determined from a software from Perkin-Elmer. Component relative concentrations were calculated based on GC peak areas without using correction factors.

### Gas chromatography–mass spectrometry

Samples were analyzed with a Perkin–Elmer Turbo mass detector (quadrupole), coupled to a Perkin–Elmer Autosystem XL, equipped with the fused-silica capillary columns Rtx-1 and Rtx-Wax (ion source temperature 150°C; energy ionization 70 eV). EI mass spectra were acquired over the mass range 35–350 Da (scan time: 1 s). Other GC conditions were the same as described under GC except split 1/80.

### Component identification

Identification of the components was based (i) on the comparison of their GC retention indices (RI) on non polar and polar columns, determined relative to the retention time of a series of n-alkanes with linear interpolation, with those of authentic compounds or literature data [33,34]; and (ii) on computer matching with commercial mass spectral libraries [33-35] and comparison of spectra with those of our personal library.

### Component quantification

Quantification of essential oil components was expressed using relative concentration in g/100 g of essential oil. The procedure included the calcul of FID response factors (RFs) relative to an internal standard. We carried out a methodology reported in the literature [36] and improved in our laboratory [37]. The application of this analytical procedure allowed the determination of the oil component relative concentrations expressed in g/100 g of essential oil. Relative amounts of individual components obtained during HS-SPME experiments, were calculated on the basis of their GC peak areas on the Rtx-1 capillary column, without FID response factor correction.

### Bacterial and yeast strains and media

The bacterial strains used in this study, i.e. *Staphylococcus aureus,Bacillus subtilis*, *Pseudomonas aeruginosa*, *Enterococcus faecalis*, *Listeria monocytogenes*, *Bacillus cereus* (Gram positive), *Escherichia coli* and *Klebsiella pneumoniae* (gram negative) were isolated at the Medical Reanimation Department of the Hospital University Center of Tlemcen in Algeria. The yeast *Candida albicans* was isolated at the Dermatology Department of the same hospital. Bacterial strains preserved in nutrient agar at 4°C, were revivified in nutrient solution and incubated at 37 ± 1°C during 18 to 24 h. 0.1 mL of each culture was added to 10 mL BHIB (Brain Heart Infusion Broth, pronadisa Hispanalab). *C. albicans* preserved at 4°C in the Sabouraud agar supplemented with chloramphenicol was revivified in nutrient solution and incubated at 30 ± 1°C during 24 to 48 h. 0.1 mL of each culture was added to 10 mL sterile physiological water. For antimicrobial assay, bacterial strains were grown on Mueller-Hinton Agar (MHA, Pronadisa Hispanalab) while *C. albicans* was grown on Sabouraud Dextrose Agar + chloramphenicol (SDA, Merck). Bacterial and yeast inoculate reached microbial densities in the range 106 to 107 cfu/mL.

### Antimicrobial activity

#### Paper-disc diffusion method

Antibacterial activities of essential oil from root and all aerial parts of the plant were assessed using the paper disk agar diffusion method according to Rios [38]. Absorbent disks (Whatman disk 6-mm diameter) were impregnated with 20 μl of oil, to concentration of 5 mg mL-1, and then placed on the surface of inoculated plates (90 mm) and incubated at 37°C for 24 h. Negative controls were prepared using a disk impregnated with the same solvent as that used to dissolve the plant oils. Antimicrobial activity was assessed by measuring the inhibition zone. All the tests were performed in triplicate.

#### Dilution-agar method

A dilution agar method was used to determine the Minimum Inhibitory Concentrations (MIC). Stock solutions were obtained by dissolving extracts in dimethylsulfoxide (DMSO 1%). Serial dilutions were made to obtain concentrations ranging from 0 to 100 μg mL-1 of the essential oil. Each mixture was added to Mueller–Hinton agar for bacteria [39,40]. The Petri dishes contained a sterile solution of DMSO and the culture medium, respectively. After incubation at 37°C for 24 h for bacteria and at 30°C for 48 h for the yeast. The experiments were performed in triplicate.

## Competing interest

The authors declare that they have no competing interests.

## Authors’ contributions

MAD performed in collection of plant material. MAD, ND and JMD performed the HD and HS-SPME extractions, obtained the essential oils and the volatiles fractions, as well as participated in the data analysis. MAD, HA, BT, AM and JC conceived the study and helped draft the manuscript. HA, BT, AM and JC performed the coordination of the study, worked on the data analysis and interpretation. All authors read and approved the final manuscript.
